# Technological Innovations and Circular Economy in the Valorization of Agri-Food By-Products: Advances, Challenges and Perspectives

**DOI:** 10.3390/foods14111950

**Published:** 2025-05-30

**Authors:** Carlos A. Ligarda-Samanez, Mary L. Huamán-Carrión, Wilber Cesar Calsina-Ponce, Germán De la Cruz, Dante Fermín Calderón Huamaní, Domingo J. Cabel-Moscoso, Antonina J. Garcia-Espinoza, Reynaldo Sucari-León, Yolanda Aroquipa-Durán, Jenny C. Muñoz-Saenz, Mauricio Muñoz-Melgarejo, Enoc E. Jilaja-Carita

**Affiliations:** 1 Nutraceuticals and Biomaterials Research Group, Universidad Nacional José María Arguedas, Andahuaylas 03701, Peru; huamancarrionmary@gmail.com (M.L.H.-C.); wcalsina@unap.edu.pe (W.C.C.-P.); german.delacruz@unsch.edu.pe (G.D.l.C.); dante.calderon@unica.edu.pe (D.F.C.H.); jesus.cabel@unica.edu.pe (D.J.C.-M.); antonina.garcia@unica.edu.pe (A.J.G.-E.); rsucari@unah.edu.pe (R.S.-L.); yaroquipa@unaat.edu.pe (Y.A.-D.); jmunoz@continental.edu.pe (J.C.M.-S.); d.mmunoz@ms.upla.edu.pe (M.M.-M.); ejilaja@unap.edu.pe (E.E.J.-C.); 2 Social Sciences Faculty, Universidad Nacional del Altiplano, Puno 21001, Peru; 3 Agricultural Science Faculty, Universidad Nacional de San Cristobal de Huamanga, Ayacucho 05000, Peru; 4 Ambiental Engineering School, Universidad Nacional San Luis Gonzaga, Ica 11001, Peru; 5 Engineering and Management Faculty, Universidad Nacional Autónoma de Huanta, Ayacucho 05000, Peru; 6 Professional Nursing School, Universidad Nacional Autónoma Altoandina de Tarma, Junin 12731, Peru; 7 Ambiental Engineering School, Universidad Continental, Huancayo 12006, Peru; 8 Human Medicine Faculty, Universidad Peruana los Andes, Huancayo 12006, Peru; 9 School of Mechanical and Electrical Engineering, Universidad Nacional del Altiplano, Puno 21001, Peru

**Keywords:** emerging technology, bioeconomy, green extraction techniques, functional ingredients, microencapsulation

## Abstract

The valorization of agri-food by-products is a critical pathway toward building sustainable food systems, reducing waste, and advancing the circular economy. This review aims to identify recent advances, key challenges, and future perspectives in this field. We conducted a critical and systematic synthesis of 159 peer-reviewed studies (2019–2025) selected based on quality and thematic relevance from leading international databases. The analysis focuses on emerging technologies such as ultrasound-assisted extraction, microencapsulation, spray drying, lyophilization, deep eutectic solvents, and colloidal systems, emphasizing their efficiency in recovering bioactive compounds from agro-industrial by-products. Significant challenges include industrial scalability, economic feasibility, regulatory compliance, and consumer acceptance. This paper also discusses current applications in functional foods and nutraceuticals, outlining promising directions for the sector. Although challenges remain, the findings offer valuable insights for researchers, industry, and policymakers aiming to foster sustainable innovation and implement strategies aligned with circular economy principles.

## 1. Introduction

Currently, the agri-food industry generates enormous quantities of by-products that are discarded or not used properly. These wastes include shells, seeds, effluents, fibrous materials, and other types of by-products, which accumulate and represent important losses of economic resources for companies and are considered an environmental problem [1,2,3]. In this context, transforming these by-products into high-value functional and advanced products emerges as an important alternative within the bioeconomy and circular economy fields that seek to minimize waste and promote the competitiveness and sustainability of value chains [4,5,6].

In recent years, different technological innovations have been developed to generate added value from different agri-food waste, such as ultrasound-assisted extraction, encapsulation, spray drying, freeze-drying, extraction with supercritical carbon dioxide, enzymatic hydrolysis, controlled fermentations, microwave and vacuum drying, and the development of bioplastics through biotechnology and 3D printing [2,7,8,9,10,11,12,13,14]. Digital technologies have recently emerged in the agri-food industry through biosensors, artificial intelligence (AI), big data, and blockchain. These are considered modern tools for optimizing agri-food processes, guaranteeing traceability, and improving the sustainability and competitiveness of production chains [15,16,17].

Many of these technologies have been successfully applied to recover antioxidants, pigments, polyphenols, proteins, and dietary fiber, among other compounds. Several studies highlight that these compounds are even present in higher concentrations in the discarded parts than in the marketed ones, reinforcing their importance [2,8,18] for utilization. These technologies have contributed to developing functional foods, various supplements, nutraceutical ingredients, and biodegradable packaging from agro-food by-products [19,20,21,22,23,24,25,26,27]. However, challenges related to scalability, process standardization, cost feasibility, and consumer acceptability of ingredients derived from various wastes persist [28,29,30,31]. It is important to mention that although this review article presents a broad overview of currently available technological innovations, the analysis focuses on those related to bioactive compounds’ extraction, encapsulation, and functional application due to their relevance and potential.

Recently, interest has grown in using agro-industrial by-products as functional ingredients in diverse food matrices, expanding their application beyond traditional uses. By-products such as fruit pomace, oilcake, and vegetable peels are increasingly appreciated not only for their content of polyphenols, anthocyanins, dietary fiber, and vitamins but also for their ability to replace synthetic additives in food formulations. Their incorporation into sorbets, dairy products, emulsions, and meat analogs has improved nutritional value, oxidative stability, and sensory appeal. This broader perspective underscores the untapped potential of many by-products, particularly when combined with modern extraction and formulation techniques. Their use supports more natural, health-oriented innovations and aligns with the principles of a circular and sustainable economy [3,32,33,34,35,36].

**Figure 1:**
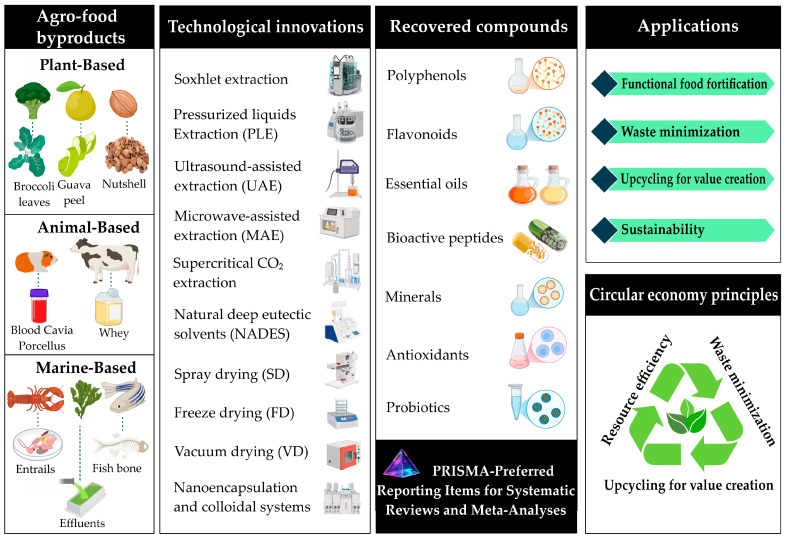
General scheme for valorizing agri-food by-products through technological innovations in the circular economy context.

Unlike previous reviews, which focused on individual technologies or partial aspects of the valorization of agri-food by-products, this review article offers an integrated and critical view of the main technological innovations framed within the bioeconomy and the circular economy, considering not only the technical aspects but also the regulatory, economic, and social challenges. In addition, this article makes a rigorous and updated selection of the relevant literature. Thus, the study synthesizes existing knowledge and identifies key gaps in scalability, consumer acceptance, and regulatory frameworks, providing necessary insights to guide future research and facilitate industrial adoption of these sustainable approaches. The article also provides a multidimensional analysis that connects technological innovations with their regulatory, economic, and social implications, establishing a direct bridge between academic developments and their industrial applicability. A general outline that guided the development of the review manuscript is shown in Figure 1.

## 2. PRISMA (Preferred Reporting Items for Systematic Reviews and Meta-Analyses) Methodology

We used a structured approach following the PRISMA methodology for this review article to ensure a systematic, transparent, and reproducible search. For this purpose, databases such as Scopus, ScienceDirect, SpringerLink, Taylor & Francis, MDPI, Wiley, and PubMed were thoroughly searched, considering studies published between 2019 and 2025 [37,38,39]. Initially, articles related to the valorization of agri-food by-products through technological innovations were identified. After eliminating duplicate records, titles and abstracts were reviewed, excluding those not meeting the inclusion criteria. Ultimately, 159 articles were selected for this paper’s analysis and critical synthesis. Although a formal analysis of publication bias was not conducted, efforts were made to include high-quality and scientifically relevant studies covering original experimental articles and recent reviews that provided a comprehensive overview of the selected research field.

## 3. Fundamentals of the Valorization of Agri-Food By-Products

The bioeconomy and the circular economy promote key sustainable models for valorizing agri-food by-products. In this context, a clear distinction is made: wastes generally lack immediate utility and are often discarded, while by-products are secondary materials generated during processing that retain functional, nutritional, or technological potential. These by-products can be exploited by applying emerging technologies in different industries [8,40,41,42]. These by-products depend on the origin of the raw materials and the technological process applied; the most common are hulls, seeds, bagasse, remaining pulp, leaves, roots, and liquid effluents from fruit, cereal, oilseed, meat, and dairy industries. It is known that they have historically been underutilized but contain valuable bioactive compounds such as phenolic compounds, fibers, proteins, functional lipids, minerals, and natural pigments. By taking advantage of these, the efficiency of agro-food processes could be improved, thus reducing the negative environmental impact [43,44]. It is known that the principles of the circular economy allow the redesign of processes to minimize waste and thus maintain the value of resources for as long as possible. In a complementary manner, the bioeconomy promotes different wastes to generate added value, as in the case of ingredients and diverse materials [45,46,47,48,49].

In addition, it is important to note that strategically utilizing biodiversity and underutilized crops, especially in developing countries, is also necessary. These resources include Indigenous tubers, underutilized cereals, wild fruits, livestock residues, and biopolymer-rich structural residues, which emerging technologies could exploit. Their incorporation into value chains can improve local competitiveness, enhance food security, and conserve agrobiological heritage [50,51,52,53,54,55,56,57].

The challenges are diverse and must be addressed, considering the diversity of each production sector and complying with international and national regulations on safety and traceability. In addition, the potential of biomass to generate energy and advanced biomaterials with different uses must be recognized. Reaching this level of application requires prior in-depth characterization studies. On the other hand, the inclusion of underutilized native crops in value chains generates economic and environmental benefits, ensuring a positive social impact on sustainable development. Based on these fundamentals, it is possible to apply emerging technologies that allow these by-products to be used efficiently, as detailed in the following section.

**Figure 2:**
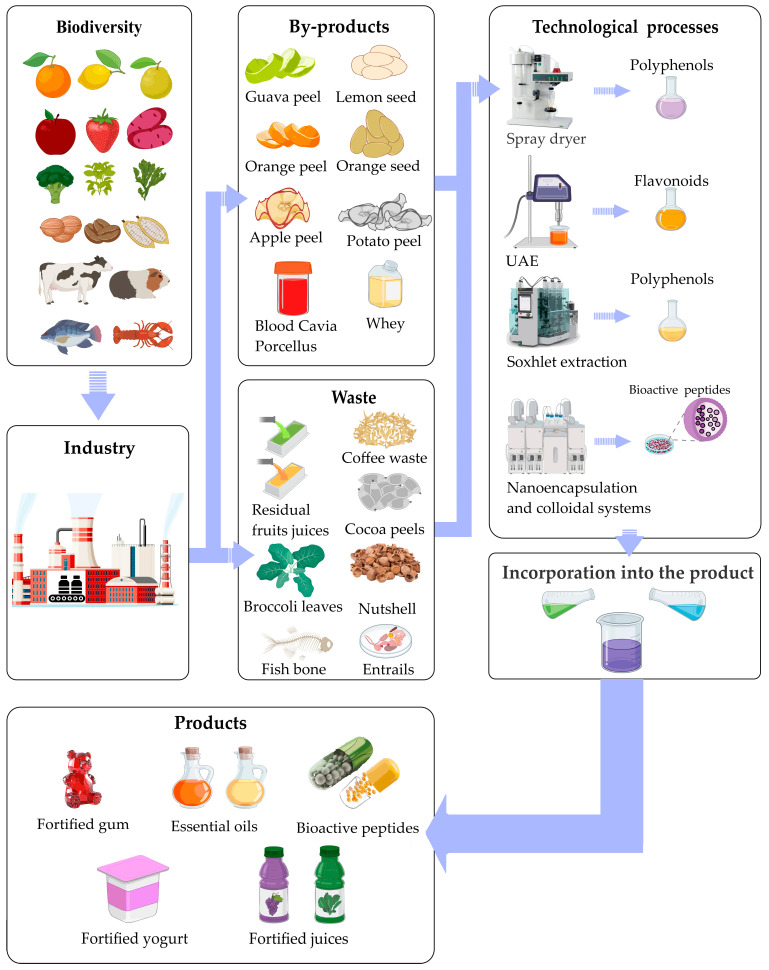
Fundamentals of the valorization of agri-food by-products.

In this section, not only were conventional industrial by-products considered, but the analysis was also extended to indigenous and underutilized crops, highlighting their strategic relevance within the bioeconomy. Figure 2 shows the rationale for the valorization of agri-food by-products.

## 4. Technological Innovations Applied to the Processing of Agri-Food By-Products

New advances are being developed worldwide that make it possible to improve technological processes, making them more efficient, sustainable, and compatible with the principles underpinning the bioeconomy and circular economy described in the previous section. Through the use of these emerging technologies, it is possible to transform agri-food by-products into innovative value-added products [8,58,59]. Among the most prominent technologies are those that allow the extraction, purification, encapsulation, and release of bioactive compounds, which, once validated, can be used in different food matrices [60,61,62,63,64]. The following subsections describe the most important advances in this field.

### 4.1. Extraction and Recovery of Bioactive Compounds

The efficiency in the extraction of bioactive compounds directly affects the yield and functional quality of the extract obtained. Likewise, recovering these compounds from agro-industrial by-products is an important phase for valorization within the circular economy framework [65,66,67]. The choice of the most appropriate technique has a decisive influence on preserving the structure of compounds sensitive to heat and oxidation, such as polyphenols, carotenoids, flavonoids, and essential oils [43]. Therefore, traditional methods, emerging technologies, and combined strategies have been developed and applied to improve the recovery of these target metabolites [8,34]. The most commonly used extraction techniques are traditional methods, pressurized liquid extraction, ultrasound-assisted extraction, microwave-assisted extraction, supercritical carbon dioxide extraction, natural deep eutectic solvents, and other strategies combining methods [34,68,69,70]. Table 1 compares the most important extraction techniques, detailing their principle of operation, advantages, disadvantages, and the various types of bioactive compounds that can be recovered, taking into account the most up-to-date scientific information.

The choice of extraction technique depends primarily on the type of by-product, the target bioactive compound, and the technological context. For example, methods such as ultrasound-assisted extraction (UAE), mi-robe-assisted extraction (MAE), and supercritical CO_2_ extraction (SFE) offer high efficiency and selectivity but present challenges in cost, scale-up, and standardization [33,35,80,82,83,84,85,86,87,88,89,104]. In contrast, traditional methods are less sustainable but are still used because of their operational simplicity and low cost. In the case of natural eutectic deep eutectic solvents (NADES) and their combinations with methods such as UAE or MAE, they emerge as promising alternatives within the framework of green extraction. However, they still require industrial validation due to challenges such as high viscosity and the recovery of the extracted compound [93,94,95,96,97,102]. This comparison highlights that there is no universally superior technique; the optimal selection must carefully consider matrix characteristics, extraction objectives, and specific requirements for scale-ability and industrial implementation, aligning with a strategic approach to compound recovery that adds value to agro-industrial by-products under the principles of sustainable development.

After obtaining the extracts from the agro-industrial by-products and techniques described above, it is sometimes possible to carry out a subsequent purification or concentration of the bioactive compounds obtained. This stage may include ultrafiltration, selective precipitation, or chromatography techniques, improving the extracts’ quality, stability, and functionality before their subsequent encapsulation [34,65,66]. However, taking into account the focus on sustainability and minimization of additional steps in the valorization of agro-industrial by-products, this review article addresses advances in encapsulation and stabilization applied directly to crude or poorly purified extracts, as proposed in recent advances for the utilization of by-products [8,40,43].

### 4.2. Encapsulation and Stabilization

Both strategies are key because they preserve the functionality of bioactive compounds against adverse conditions such as oxidation, light, heat, or pH. These techniques improve stability during storage, reduce undesirable interactions with other food components, and allow controlled release of bioactive compounds. Within the valorization of agro-industrial by-products, encapsulation and stabilization have become an efficient alternative for protecting phenolic compounds, carotenoids, essential oils, bioactive peptides, probiotics, and other compounds [8,18,40,43,105].

The most commonly used encapsulation techniques are spray drying, freeze drying, and vacuum drying. Spray drying is one of the most widely applied methods on an industrial scale due to its low cost, scalability, and ability to generate dry particles in a single step. However, thermostable materials are required because high temperatures are reached during drying [106,107,108]. On the other hand, freeze-drying allows better preservation of the structure of heat-sensitive compounds. However, its application is limited by its high energy consumption and the longer time required [109,110]. In the case of vacuum drying, this represents an intermediate option, with moderate temperatures and less impact on the encapsulated compounds [111,112].

Recently, nanoencapsulation techniques and colloidal systems such as multiple emulsions or polymeric nanoparticles have been developed. These emerging technologies allow obtaining particles smaller than 1000 nm, improving dispersion in aqueous media, increasing bioavailability, and favoring a controlled release in food matrices [113,114]. In addition, colloidal systems stabilized by polysaccharides or proteins effectively protect hydrophobic compounds from environmental degradation [115,116]. Forming nanoemulsions or colloidal systems is often a prerequisite for encapsulation, especially when dealing with hydrophobic bioactive compounds, as it enhances dispersion and encapsulation efficiency [113,114,115,116].

The selection of the encapsulating material is a determining aspect of the system’s efficacy. Polysaccharides such as alginate, starch, maltodextrin, gum arabic, tara gum, and chia mucilage, among others, are widely used for their biocompatibility and ability to form gels and protective films [8,50,117,118]. Proteins (gelatin, casein, and whey protein) are also used as encapsulating agents, especially when hydrophobic interactions with bioactive compounds are required [119,120]. In some formulations, materials are combined to improve encapsulation efficiency and stability, as in alginate-chitosan or polysaccharide-protein systems [121,122,123].

Additionally, food formulations’ encapsulating materials and bioactive ingredients must comply with food safety regulations. In this context, several substances must hold the Generally Recognized as Safe (GRAS) status in the United States or equivalent approvals in other jurisdictions, ensuring their safe incorporation into functional food systems [7,28,40].

Encapsulation’s stability against environmental factors depends on the nature of the wall material, particle size, drying method, and storage conditions. Encapsulates may experience losses due to oxidation, hydrolysis, or agglomeration, especially in the presence of moisture or light. However, several strategies have shown positive results in improving stability, such as using double coatings, ionic gelation, or matrix-integrated antioxidative agents [8,117,124,125].

Finally, one of the most relevant attributes of encapsulation is the ability to control the release of the encapsulated bioactive compound. Controlled-release systems enable the bioactive compound to be directed to a specific site in the gastrointestinal tract or released gradually during processing or storage, thereby increasing its functional efficacy. Sustained release profiles have been reported using pH-sensitive materials, multilayers, or biodegradable matrices [8,40,43,124,126,127].

These approaches show that selecting the technique and encapsulating materials should be strategic, considering the type of bioactive compound, the matrix, and the specific functional targets, as no single superior method is universally applicable. Table 2 summarizes the primary encapsulation methodologies, considering their principle of operation, encapsulating materials, encapsulated bioactive compounds, advantages, and limitations based on the reviewed literature applied to this context.

## 5. Application in the Development of New Products

The valorization of agri-food by-products through various technologies has allowed their strategic incorporation in developing value-added foods. This practice is aligned with the principles of the circular economy. Bioactive compounds such as polyphenols, flavonoids, soluble fibers, carotenoids, bioactive peptides, and minerals can be extracted, purified, and encapsulated from peels, seeds, pulps, leaves, or effluents of fruits, vegetables, and other underutilized crops [8,40,43,54,143].

One of the most relevant applications is fortifying food products by adding extracts or microencapsulates from by-products. These incorporations can be made in dry powder form, as colloidal emulsions, or using encapsulated systems that protect the compounds against environmental factors and release their content in a controlled manner in the gastrointestinal tract (GIT) [111,115,117].

From a nutritional perspective, several studies have shown that including agro-industrial by-products improves the content of dietary fiber, antioxidants, and other beneficial nutrients without significantly altering the food’s sensory properties. It has also been observed that encapsulated compounds can improve techno-functional properties such as water retention, thermal stability, and antimicrobial activity, thanks to the joint action of the bioactive compounds and the materials used as encapsulating agents, such as starches, gums, or vegetable fibers [36,117,124].

**Figure 3:**
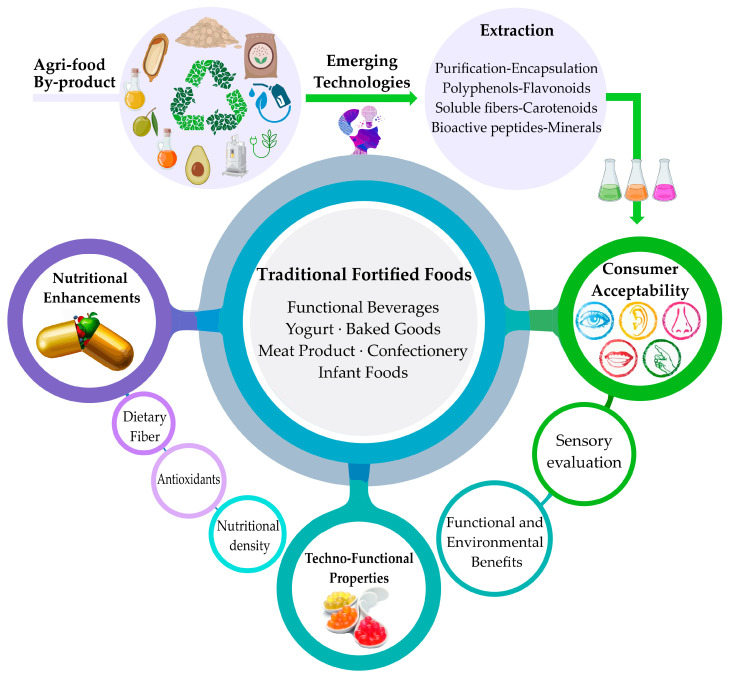
Route for the valorization of agri-food by-products for the development of functional foods.

On the other hand, sensory evaluation has become particularly relevant since consumers may resist products made with ingredients derived from residues. However, recent research indicates that, with proper microencapsulation and formulation, favorable acceptability can be achieved, especially when the functional and environmental value of the product is adequately communicated [3,28,40]. Figure 3 shows the valorization route of agro-food by-products for developing functional foods.

Several studies have demonstrated successful applications of agro-industrial by-products in real food matrices. These include yogurts fortified with encapsulated carrot waste extract [117], functional gummies made with microcapsules of guinea pig blood erythrocytes and tumbo juice [111], and edible oils stabilized with nanoencapsulated pomegranate peel extracts [115]. The use of grape pomace as a source of encapsulated bioactive compounds for controlled release in food products has also been documented [110], as well as the incorporation of vegetable by-products such as onion peel or mango pulp in functional formulations [134]. These experiences demonstrate the real potential of waste valorization as a strategy for the design of value-added foods.

In addition, successful applications have been reported that include the incorporation of fruit and vegetable wastes in bakery products, functional juices enriched with polyphenols, energy bars with coffee and cocoa by-products, and the use of vegetable extracts in dressings and soups, which further expands the possibilities for the design of functional foods based on valorized wastes [62].

Other possible uses for by-products include apple pomace, banana peel, papaya seeds, and grape skins in food products such as muffins, cookies, breads, noodles, dairy products, and beverages. These by-products are often processed by microencapsulation or nanoencapsulation methods to improve the stability and efficacy of their bioactive compounds. In addition, other applications used seaweed extracts in dairy applications, cocoa shells in extruded snacks, and cereal bran in emulsified meat formulations. As the above examples show, there is a vast potential for use, especially when using emerging technologies [36,144,145].

## 6. Current Challenges and Barriers to Industrialization

Despite emerging technologies demonstrating the potential for valorizing agri-food by-products, their industrial-scale application still faces significant technical, regulatory, and societal challenges. One of the main obstacles is scalability, as many processes have been validated only at laboratory or pilot scale and still require optimization for real production environments [28,40]. Technologies such as microencapsulation, ultrasound-assisted extraction, and spray drying often involve high capital investment, elevated energy consumption, and the need for precise control of operational parameters [8]. Moreover, the natural variability of by-products—depending on species, region, or season—complicates process standardization and hinders reproducibility across production batches [28,40]. These technical constraints, combined with the limited number of pilot-scale validations in industrial settings, restrict progress toward more sustainable production models based on the integrated use of by-products.

From a regulatory standpoint, although interest in by-product valorization is growing, significant gaps in legislation still hinder the incorporation of these materials into functional foods. In many countries, regulations concerning agro-industrial residues remain primarily focused on environmental management rather than their potential as sources of functional ingredients, creating an uncertain legal framework for industrial adoption [7,28,40,58]. Additionally, authorization processes for novel ingredients derived from by-products are often lengthy, costly, and inaccessible to small and medium-sized enterprises [7,28]. The lack of harmonized guidelines on traceability, microbiological criteria, labeling, and food safety slows adoption and limits scalability. Furthermore, existing legal frameworks rarely consider the multifunctional use of residues within integrated bioeconomy models, limiting cross-sectoral innovation [7,58].

Beyond technical and regulatory challenges, consumer perception is among the most critical and often underestimated barriers to market adoption of food products derived from agri-food by-products. Consumers frequently associate “waste” with low quality or lack of safety, leading to resistance or rejection of such products [28]. However, recent studies have shown that strategies such as microencapsulation and education and awareness campaigns can significantly improve consumer acceptability and perception [28,40]. Moreover, local and native by-products may increase perceived value, especially when linked to regional identity, sustainability, and economic development [40]. On the other hand, the absence of transparent and accessible information regarding the origin and processing of ingredients can fuel consumer mistrust, reinforcing the need for clear communication to support sensory acceptance and purchasing decisions [58,146].

Altogether, these interrelated challenges underscore that the success of technological innovations for by-product valorization depends not only on scientific and technical progress but also on regulatory evolution, inclusive economic models, and the social acceptance of novel food ingredients. These elements are essential to define realistic perspectives and promote broader industrial adoption in line with circular economy principles.

## 7. Future Perspectives and Trends

Future trends in the valorization of agri-food by-products are oriented toward combining emerging technologies to enhance process efficiency and sustainability. The integrated use of ultrasound-assisted extraction, green solvents, microencapsulation, and colloidal systems has shown significant improvements in the recovery and stability of bioactive compounds, enabling their incorporation into functional food matrices [110,111,112,113,114,147,148]. In particular, nanoencapsulation and nanoemulsions are emerging as key tools to increase the bioavailability, protection against adverse conditions, and controlled release of antioxidants, polyphenols, and bioactive peptides [113,114]. These advances improve functional and nutritional properties and enable the development of more innovative, personalized products tailored to consumer health demands [8,28,40,58]. Furthermore, future research is expected to optimize encapsulating materials (such as chitosan, alginate, and starch nanoparticles) to develop innovative systems capable of responding to specific stimuli such as pH, temperature, or intestinal microbiota, thus broadening industrial and biomedical applications [8,28,40,50,56]. While nanoencapsulation and nanoemulsions stand out for their high efficiency and functional potential, their high costs hinder scalability in emerging regions. In contrast, spray drying and freeze-drying, although energy-demanding, are more widely accepted in various regions worldwide, making it necessary to analyze costs and the specific regional context.

Other emerging technologies, such as pulsed electric fields, cold plasma, and precision fermentation, are gaining interest due to their potential to process agri-food by-products with low energy consumption and high compound selectivity. Although still under development, these approaches may complement existing methods by enabling new ways to extract, transform, or stabilize bioactive compounds within sustainable and scalable frameworks [31,143].

The industrial integration of agri-food by-product valorization technologies requires advancing toward collaborative and scalable models that bridge academic developments with the real demands of the production sector. The establishment of pilot plants and the execution of semi-industrial scale studies are essential to validate processes mainly developed in laboratory settings [28,40]. Additionally, the importance of promoting partnerships between universities, research centers, companies, and local governments is emphasized to accelerate the adoption of technological innovations in the food industry. Such collaborations enable resource and capacity sharing, strengthen regional value chains, generate positive economic impacts in rural communities, and foster the bioeconomy. Moreover, applying circular economy indicators to the agro-industrial sector allows for monitoring and managing these initiatives, ensuring that the environmental, economic, and social benefits derived from agro-industrial by-product valorization are maximized [8,28,40,58,149,150].

From a social and commercial standpoint, future perspectives in the valorization of agri-food by-products focus on improving consumer perception and reinforcing trust in these products. Applying science-based communication strategies that clearly explain the nutritional, environmental, and economic benefits is key to increasing acceptance and willingness to pay for such foods [32,147,151,152,153,154]. Additionally, specific certifications, sustainability labels, and seals that highlight these products’ circular and eco-friendly nature can favorably differentiate them in the market, enhancing their appeal. Global trends show that consumers, especially younger generations, increasingly demand functional and sustainable foods aligned with circular economy principles, representing a strategic opportunity to expand these innovations [8,28,58,155,156,157,158,159].

**Figure 4:**
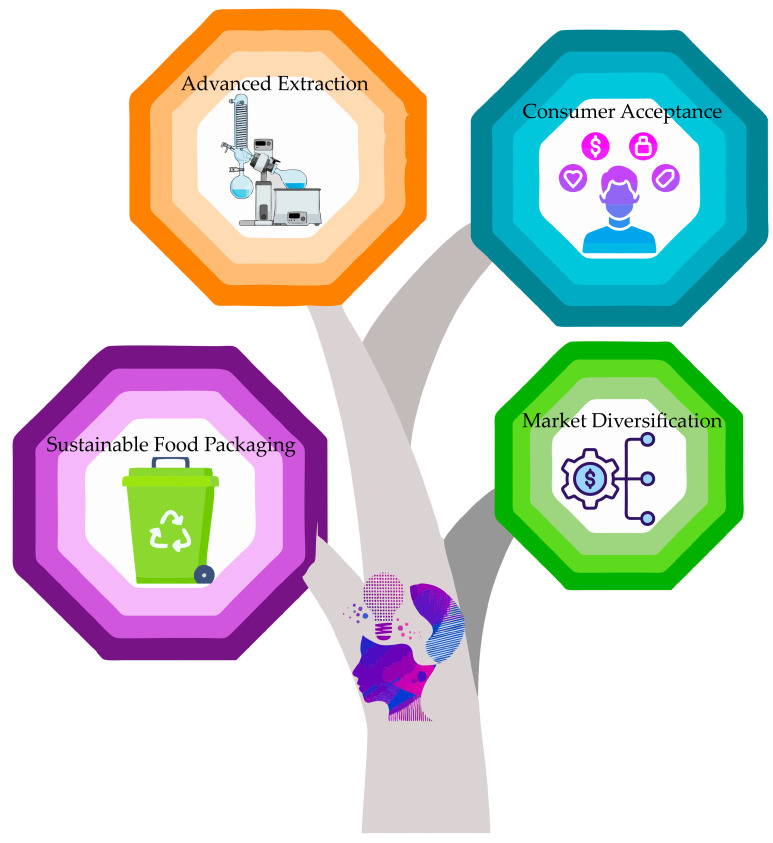
Future trends in the valorization of agri-food by-products through emerging technologies.

Finally, from a regulatory and policy perspective, strengthening the frameworks that enable the safe and efficient incorporation of ingredients derived from agri-food by-products into production chains is essential. Harmonizing regulations, developing supportive public policies, and creating specific incentives can significantly accelerate the adoption of these approaches, ensuring positive environmental and economic impacts. Figure 4 illustrates the future trends in the valorization of agri-food by-products through emerging technologies.

## 8. Conclusions

This review provides an integrated synthesis and analysis of the main technological innovations in valorizing agri-food by-products within the bioeconomy and circular economy framework. It highlighted the potential of emerging technologies such as ultrasound-assisted extraction, microencapsulation, spray drying, deep eutectic solvents, nanoencapsulation, and colloidal systems, all of which are capable of recovering high-value bioactive compounds, generating new functional ingredients, and reducing environmental impact. In addition, critical challenges related to scalability, costs, regulatory frameworks, and consumer perception were identified, which currently limit their large-scale application.

Based on the findings, future research is recommended to prioritize pilot- and industrial-scale validation of these technologies, incorporating detailed economic analyses, consumer perception studies, environmental impact assessments, and life cycle approaches. Developing harmonized regulatory frameworks and communication strategies that enhance social acceptance of products derived from agri-food by-products is also necessary, strengthening their integration into sustainable value chains.

This study has some limitations that should be considered. The heterogeneity of the analyzed studies, in terms of methodologies, types of by-products, and technologies, makes it difficult to establish direct quantitative comparisons between the results. Furthermore, although a systematic search was conducted, it was impossible to include all the available literature. Finally, in-depth life cycle analyses, environmental impacts, or detailed economic models were not addressed, representing relevant opportunities for future work.

## Figures and Tables

**Table 1:** Comparative characteristics of extraction techniques applied to agro-industrial by-products.

Extraction Technique	Principle	Advantages	Disadvantages	Types of Recovered Bioactive Compounds	Agri-Food By-Products and Waste (Examples)	References
Traditional methods (Soxhlet extraction, maceration, infusion, etc.)	Based on heat-driven diffusion using conventional organic solvents.	Simple operation, low cost, and widely established techniques.	Use of toxic solvents, low efficiency, and long extraction times.	Polyphenols, essential oils, tannins, anthocyanins.	Guava leaves, potato peel, acerola waste, green walnut husks.	[71,72,73,74,75]
Pressurized liquid extraction (PLE/ASE)	Involves liquid solvents at elevated temperature and pressure to improve diffusion and solubility of bioactive compounds.	Fast, automatable, and efficient for extracting polar and phenolic compounds, with reduced solvent consumption.	Expensive equipment, potential thermal degradation if temperature is not controlled.	Phenolic acids, flavonoids, lignans, alkaloids, antioxidant compounds.	Grape seed, coffee silverskin, olive leaves, grape pomace, seaweeds.	[65,76,77,78,79]
Ultrasound-assisted extraction (UAE)	Ultrasound generates acoustic cavitation, enhancing solvent penetration and facilitating cell disruption.	High efficiency, low solvent consumption, suitable for thermolabile compounds.	Challenges for industrial scalability, potential degradation due to intense cavitation.	Polyphenols, flavonoids, carotenoids, essential oils.	Pistachio oilcakes, grape pomace, red lobster by-products, citrus waste, strawberry by-products.	[33,34,35,80,81,82,83]
Microwave-assisted extraction (MAE)	Microwaves generate electromagnetic fields that interact with polar molecules, producing internal heating.	Fast, efficient, and energy-saving.	Requires precise temperature control; some equipment is expensive.	Phenolic compounds, carotenoids, essential oils.	Opuntia cladodes, pomegranate peel, broccoli leaves, spent coffee grounds, mango peel, lemon peel.	[84,85,86,87,88]
Supercritical CO_2_ extraction (SFE)	Involves the use of CO_2_ in a supercritical state as a green, non-toxic, and recyclable solvent.	High selectivity, no toxic residues, clean extraction.	High initial investment, limited efficiency for polar compounds.	Carotenoids, tocopherols, lipophilic bioactives, antioxidants.	Rosehip shells and seeds, hop cones, *Berberis microphylla*, rowanberry pomace.	[89,90,91,92]
Natural deep eutectic solvents (NADES)	Formed by combining hydrogen bond donors and acceptors to produce eutectic liquids.	Eco-friendly, biodegradable, customizable for specific compounds.	High viscosity, complex recovery of extracted compounds.	Alkaloids, polyphenols, flavonoids, terpenoids.	Wild thyme, perilla leaves, orange peel, coffee grounds, citrus peel waste, hazelnut by-products.	[93,94,95,96,97,98]
UAE combined with NADES	Combines acoustic cavitation with natural deep eutectic solvents to enable selective extraction.	High efficiency, low toxicity, low-temperature extraction, good selectivity depending on HBA-HBD (hydrogen bond acceptor/donor), useful in plant matrices.	High NADES viscosity, solute recovery may require SPE or lyophilization; scalability challenges.	Polyphenols, flavonoids, anthocyanins, carotenoids (astaxanthin), asiaticoside, oleuropein, lignin, modified pectin, betalains.	Blueberry pomace, blueberry peel, blueberry wine residues.	[68,69,99,100,101,102,103]

Note: The examples listed under “agri-food by-products and wastes” include secondary materials with the added value generated during primary processing (by-products) and food processing wastes commonly discarded but valorized. Some references were included based on their methodological relevance to describe extraction principles, compound recovery, or technological considerations.

**Table 2:** Encapsulation and stabilization technologies are applied to valorizing bioactive compounds from agri-food by-products.

Technique/Method	Operating Principle	Encapsulating/Stabilizing Materials	Encapsulated Compounds	Key Advantages	Main Limitations	References
Spray Drying (SD)	Atomization of liquid mixtures into a hot air stream to produce a powder in a single step.	Maltodextrin (cassava starch), inulin (chicory root), gum arabic (*Acacia senegal*), trehalose (maize starch), soy protein isolate (soybean meal), β-cyclodextrin (corn starch), starch (cassava), tara gum (*Caesalpinia spinosa*), mucilage (chia, basil, cactus seeds, pitahaya peel)	Punicalagin, lycopene, β-carotene, polyphenols, flavonoids, anthocyanins, heme iron.	Cost-effective, scalable, good powder stability, suitable for heat-stable compounds	Thermal degradation of heat-sensitive compounds	[56,106,107,128,129,130,131,132,133,134]
Freeze Drying (FD)	Freezing followed by sublimation of water under vacuum conditions.	Maltodextrin (cassava starch), gum arabic (*Acacia senegal*), whey protein (bovine milk), gelatin (porcine or bovine collagen), casein (skim milk), β-cyclodextrin (corn starch), carboxymethylcellulose (plant cellulose).	Lycopene, flavonoids (naringin, naringenin), anthocyanins.	Excellent preservation of thermosensitive compounds, formation of porous and lightweight matrices.	High operational cost, time-consuming process.	[8,109,110,131,135,136,137]
Vacuum Drying (VD)	Water removal under low pressure and temperature.	Maltodextrin (cassava starch), inulin (chicory root), tara gum (*Caesalpinia spinosa*).	Anthocyanins, polyphenols, flavonoids, heme iron.	Preservation of organoleptic properties, reduced energy consumption.	Slower drying rate compared to spray drying.	[111,112,138]
Nanoencapsulation (Emulsion/Colloidal)	Formation of emulsions or colloidal nanostructures (<1000 nm) through high-energy methods such as high-pressure homogenization, ultrasonication, or high-shear mixing.	Chitosan (crustacean shells), soy protein (soybean meal), alginate (brown seaweed), starch nanoparticles (cassava or corn starch), chitin nanofibers (insect or crustacean exoskeletons)	Polyphenols, curcumin, lycopene.	High bioavailability, targeted release, antioxidant protection.	Complex formulation, sensitivity to pH and temperature.	[113,114,115,116]
Ionic Gelation	Formation of hydrogels via ionic cross-linking between anionic polymers and divalent cations such as Ca^2^^+^.	Sodium alginate (brown seaweed).	Polyphenols, β-carotene, hydrolysable tannins, protein hydrolysates.	Efficient taste masking, antioxidant and antimicrobial protection, high encapsulation efficiency, and storage stability.	Limited structural stability under uncontrolled humidity or high ionic strength conditions	[117,118,121,139]
Coacervation Encapsulation	Phase separation of polymers through electrostatic interactions between oppositely charged biopolymers.	Whey protein isolate (bovine milk), pectin (apple or citrus peel), gelatin (porcine or fish skin), gum arabic (*Acacia senegal*), sodium alginate (brown seaweed).	Phenolic compounds, anthocyanins.	Formation of monodisperse particles, good initial stability, antioxidant protection, simple and eco-friendly technique.	Sensitive to extreme pH and ionic strength conditions.	[8,140,141,142]
Extrusion Encapsulation	Dropping the core solution into a calcium chloride (CaCl_2_) gelling bath to form hydrogel beads.	Sodium alginate (brown seaweed), chitosan (crustacean shells).	Carotenoids, polyphenols, probiotics.	Simple and economical technique, provides good protection for probiotic viability.	Produces large particle sizes; less suitable for clear or transparent liquid matrices.	[8,117,122]

Note: This table summarizes the techniques to encapsulate bioactive compounds derived from agri-food by-products. Wall materials are listed with their origin in parentheses. The cited references support their application and methodological relevance.

## Data Availability

No new data were created or analyzed in this study. Data sharing is not applicable to this article.

## Abbreviations

The following abbreviations are used in this manuscript:

3D	Three-Dimensional
AI	Artificial Intelligence
ASE	Accelerated Solvent Extraction
FD	Freeze Drying
GIT	Gastrointestinal Tract
GRAS	Generally Recognized As Safe
HBA	Hydrogen Bond Acceptor
HBD	Hydrogen Bond Donor
MAE	Microwave-Assisted Extraction
NADES	Natural Deep Eutectic Solvents
PLE	Pressurized Liquid Extraction
PRISMA	Preferred Reporting Items for Systematic Reviews and Meta-Analyses
SD	Spray Drying
SFE	Supercritical Fluid Extraction
UAE	Ultrasound-Assisted Extraction
VD	Vacuum Drying
